# Remarks on Scarlet Fever

**Published:** 1833

**Authors:** George E. Conant

**Affiliations:** Windham, Portage County, Ohio


Art. II.—*Remarks on Scarlet Fever, in the Northeast part of Ohio.*

Windham, *Portage County, Ohio, March 20th*, 1833.

*To the Editor of the Western Journal.*

Sir: Having seen some notices of the Scarlet Fever, in your valuable periodical, and the views there contained, with regard to the treatment, not agreeing with my practice and experience, I have been induced to submit to your consideration, the outlines of my management, with a few observations, which you are at liberty to lay before your readers; and if any thing shall be offered that may lead to a more successful treatment of the disease, I shall have the pleasure of having subserved the interests of community.

About a year ago, the scarlet fever, for the first time, made its appearance in this place and vicinity. Of its ætiology, I can say little. From its appearance and progress, I am decidedly of the opinion that it is not contagious, but epidemic. It prevailed generally in fair weather, immediately succeeding rains, particularly if it cleared off warm and dry. After the equinoctial storm in September, it appeared within three hours of the same time, at five or six different places, in the circuit of fifteen miles.

Those cases which came under my practice, were generally of the anginose kind, though some were of a mild, and some of a more malignant character. It prevailed mostly among children, though there were a few cases among adults. The fever was generally ushered in with cold chills, high arterial action and excessive heat of the surface, in which cases, the rash made its appearance, sometimes in three or four hours, while in others it delayed as many days. There was swelling of the tonsils, uvula, and parotid glands, but no sloughing. In a short time, the tongue became glossy, and of a deep red color, also the fauces and throat put on a similar appearance. In others, the mouth was filled with canker. In summer and fore part of fall, most of the cases were taken between midnight, and six o'clock in the morning, with violent vomiting and purging.

The fever generally commenced with high excitement, but if care was not taken it soon ran into a typhus form; while a few cases put on this appearance from the beginning. In one, particularly, which succeeded immediately after the measles, the patient sunk into a low typhoid state, with cold clammy sweat. In this instance, and one other in the same family, but fever of a different grade, there was a complete cessation of voice for seven days. Generally in the commencement, the throat was sore, and deglutition difficult. This symptom lasted only about forty-eight hours. After about the third or fourth day, there was considerable pain and swelling of the wrist and ankle joints, which continued through the remainder of the disease. In some, this affection was quite tedious, and it was present in almost every case; a fact I do not recollect to have seen noticed by any author in treating of this fever. It was mitigated by the application of poultices of wheat bran and vinegar. The fever continued from three to five days; generally beginning to subside on the fourth, but never increasing longer than till the fifth day. At this stage of the disease, the desquamation of the cuticle commenced. On the fourth day the rash commonly rose up in small watery vesicles with the apex whitish; the surface having the feel of a grater; in a short time after this symptom made its appearance, the firey redness of the surface was changed to a chocolate color, and then all the unpleasant symptoms rapidly subsided.

### Treatment.

In the commencement, I prescribed a cathartic of equal parts by measure of jalap, sulphur, and cream. tartar. If the arterial action was high and the skin hot, I made use of tepid affusions to the body and extremeties, with cold water and vinegar to the head. The external application to the throat, was flannel cloths wet in a strong decoction of red pepper and vinegar. To those of the greatest severity, I made use of mustard poultices, and emplast. canthar. with benefit. Internally, the mouth and fauces were gargled frequently with a decoction of blackberry briar root, to which borax was added, and made sweet with honey. In the most severe cases, myrrh, cyenne pepper, and brandy, were made use of with the happiest effects. As expectorants, antimonial wine and vinegar of quills, were found to be of great service. But the remedy on which I placed the greatest reliance, was a powder, composed of the carb. ammonia and nitre. When the fever was the highest, and in the first stage of the disease, I gave a greater proportion of nitre, with the addition of a little ipicac.; but as the fever began to subside, the ipicac. was entirely, and the nitre gradually withdrawn; while the ammonia was increased, and camphor added, which were given freely during the remainder of the illness. Some mild aperient, such as magnesia and rhubarb or castor oil, was administered daily, if necessary, in order to keep the bowels loose and open. When the fever began to abate, I found superior advantage from the application of bass-wood leaves wilted in warm water, to the surface generally. They produced a gentle perspiration, and broke up the fever at once. As soon as the febrile action was gone, a decoction of cinchona, orange peel, and chammomile flowers, was given with advantage. For drinks, I made use of milk and water, toast and water, apple water and herb tea, as best suited the patient. Rubefacients were applied to the feet throughout the disease.

The above is a general summary of my treatment. The effects resulting from the administration of ammonia were the most salutary. It produced no unpleasant results in any case, whether given in the high excitement or collapse; and I can recommend its use to the profession with confidence, in any stage of the disease. Opium was found to be an unsafe remedy, producing determination to the brain, and stupor. From witnessing its effects in two or three instances, I found that calomel increased the soreness of the mouth and fauces, and was detrimental, unless given in the last stages of the disease, to correct a morbid sensibility of the stomach and bowels.

The sequelæ spoken of as incident to this disease, such as dropsy, suppuration of the parotid and sub maxillary glands, slight fevers, night sweats, &c. &c. I was not much troubled with. In upwards of seventy cases, but one was followed with anasarca, and that was mild. Another patient who had been subject to scrofula, was troubled with a swelling, and ulceration of the parotid glands, which was soon corrected by the use of iodine. In a few cases, there was a disagreeable discharge from the ears. This was soon relieved by injecting into them [illegible] tion of the oxy-muriate of mercury.

The above detail, is a simple narrative of facts as [illegible]

curred, coppied principally from notes taken during the progress of the disease. There were no unpleasant symptoms that did not generally yield, immediately to this treatment; and but one that proved fatal.

Yours with respect.

GEORGE E. CONANT.

DR. DRAKE.

